# Donor Mesenchymal Stem Cells Program Bone Marrow, Altering Macrophages, and Suppressing Endometriosis in Mice

**DOI:** 10.1155/2023/1598127

**Published:** 2023-07-28

**Authors:** Shutaro Habata, Ramanaiah Mamillapalli, Abdullah Ucar, Hugh S. Taylor

**Affiliations:** Department of Obstetrics, Gynecology and Reproductive Sciences, Yale School of Medicine, New Haven, CT 06510, USA

## Abstract

Endometriosis is a chronic inflammatory gynecological disorder regulated by estrogen and characterized by the growth of endometrial tissue outside the uterus. We have previously demonstrated that mesenchymal stem cells (MSCs) contribute directly to endometriosis. Here, we investigated an indirect effect; we hypothesized that MSCs may also impact the bone marrow (BM) by regulating bone marrow-derived inflammatory cells. Endometriosis was induced in mice by transplanting uterine tissue into recipient mice followed by BM transplant. Control or MSC conditioned BM was injected retro-orbitally. Direct administration of MSCs outside of the setting of BM conditioning did not alter endometriosis. Coculture of an undifferentiated macrophage cell line with MSCs in vitro led to a reduction of M1 and increased M2 macrophages as determined by fluorescence-activated cell sorting and western blot. Conditioning of BM with MSCs and transplantation into a mouse model inhibited endometriotic lesion development and reduced lesion volume by sevenfold compared to BM transplant without MSCs conditioning. Immunohistochemistry and immunofluorescence showed that MSC conditioned BM reduced the infiltration of macrophages and neutrophils into endometriotic lesions by twofold and decreased the proportion of M1 compared to M2 macrophages, reducing inflammation and likely promoting tissue repair. Expression of several inflammatory markers measured by quantitative real-time polymerase chain reaction, including tumor necrosis factor alpha and CXCR4, was decreased in the conditioned BM. Donor MSCs were not detected in recipient BM or endometriotic lesions, suggesting that MSCs actively program the transplanted BM. Taken together, these data show that individual characteristics of BM have an unexpected role in the development of endometriosis. BM remodeling and alterations in the inflammatory response are also potential treatments for endometriosis. Identification of the molecular basis for BM programing by MSCs will lead to a better understanding of the immune system contribution to this disease and may lead to new therapeutic targets for endometriosis.

## 1. Introduction

Endometriosis is a chronic inflammatory, and often debilitating, gynecological disorder that affects up to 10% of reproductive age women [1]. Endometriosis is characterized by the presence of endometrium-like tissue outside the uterus, typically in the pelvis, causing pelvic pain and infertility [2]. The most widely accepted theory for the pathogenesis of endometriosis is the implantation of endometrial tissue derived from retrograde menstruation through the fallopian tubes [3]. However, this reflux is a physiological condition that occurs in almost all women; therefore, other factors that enhance the ectopic implantation of the shed tissue and the development of endometriotic lesions are believed to be required. Endometriosis is also a systemic disease [1] and its pathogenesis is a multifactorial process resulting in alterations far beyond the pelvis [4]. It is likely that differences in immune surveillance have a critical role in determining whether displaced ectopic endometrium will develop into endometriosis as well as driving some of the systemic manifestations of the disease. A better understanding of the role of bone marrow (BM) and its programing may suggest new therapies that target the immune defects in this disease.

BM contains immune cells as well as stem cells, including hematopoietic and mesenchymal stem cells (MSCs). Altered BM function and the production of distinct inflammatory cell functions may contribute to the disease and explain many of the systemic effects of endometriosis. MSCs are multipotent cells [5–9] that, in addition to their ability to repair tissue, have pleotropic effects on the immune system [10]. Intravenous delivery of MSCs has been shown to have beneficial effects through immune modulation in multiple diseases, including graft versus host disease, systemic lupus erythematosus, rheumatoid arthritis, inflammatory bowel disease, and diabetes [11]. To date, there is no literature on MSCs and BM programing in the etiology or treatment of endometriosis.

Macrophages comprise the majority of immune cells in the peritoneal cavity and have been shown to play an essential role in the progression of endometriosis [12–14]. In women with endometriosis, macrophages are significantly increased in the peritoneal fluid, eutopic endometrium, and endometriosis lesions [15, 16]. Recent studies described the role of macrophage memory in controlling endometriosis in human as well as in mice [17]. Macrophage involvement in the development of endometriosis has been associated with loss of their phagocytic ability and other functional changes [18–20]. However, macrophages are not homogenous and have been broadly classified into two subsets: the proinflammatory or classically activated M1 phenotype and the anti-inflammatory or alternatively activated M2 phenotype [21, 22]. Altered macrophage polarization may be involved in the development of endometriosis; however, their polarization status in endometriosis is not fully characterized [23–25].

Here, we used a novel BM reprograming technique to alter BM-derived macrophages contribution to endometriosis. Treatment of BM with *in vitro* cultured MSCs altered programing of macrophages, reduced M1 macrophage infiltration into endometriosis, and led to reduced endometriosis lesion size.

## 2. Materials and Methods

### 2.1. Animals

C57BL/6 mice (8–10 weeks) and transgenic ubiquitin-GFP mice (Stock #. 004353) were obtained from Charles River Laboratories (Wilmington, MA, USA) and Jackson Laboratory (Bar Harbor, ME, USA), respectively. All mice were exposed to a 12 hr light/dark cycle (7:00 a.m.–7:00 p.m.) with food and water provided *ad libitum*. Experiments were conducted in accordance with protocols from Institutional Animal Care and Use Committee (IACUC) of Yale University.

### 2.2. BM-Derived MSCs Purification and Expansion

BM cells were extracted from wild-type or GFP mice by flushing the marrow from femurs and tibias into cold sterile DMEM/F12 (ThermoFisher Scientific, Waltham, CA) medium. Cells were filtered through a 70 *µ*M filter and centrifuged at 700 × *g* for 8 min at 4°C. Cells (5 × 10^7^) were plated in a T75 flask in 15 ml murine MSC-specific expansion medium (Mouse MesenCult Expansion Kit, Stem Cell Technologies, Cambridge, MA) containing l-Glutamine 1% (Stem Cell Technologies) and antibiotic/antimycotic solution 1% (Sigma Aldrich, St. Louis, MO). Cells are cultured at 37°C with 5% CO_2_ and atmospheric O_2_ concentration (∼20%). At the second passage, MSCs were collected and used for BM programing experiments.

### 2.3. Flow Cytometry

Cultured-MSCs mentioned above were retrieved by trypsin digestion. Cell suspensions were then incubated with mouse TruStain FcX PLUS anti-CD16/32 (BioLegend, San Diego, CA) blocking for 10 min, followed by incubation in the dark with fluorescein isothiocyanate or phycoerythrin-conjugated monoclonal antibodies anti-CD105, anti-CD29, anti-Sca-1, anti-CD45, and anti-CD34 against mouse (BioLegend) for 30 min at room temperature. Stained cells were washed with PBS, fixed in IC fixation buffer (cat. #00-8222-49, Invitrogen, Waltham, MA, USA) and fluorescence-activated cell sorting (FACS) analysis was carried out immediately on flow cytometer BD LSR II Green, (BD Biosciences, Franklin Lakes, NJ, USA). Appropriate unstained and antibody IgG isotype controls were used for setting compensation and determining gates. Data were analyzed using the software FlowJo V10.

Mouse macrophage cell line J774A.1 cells were obtained from the American Type Culture Collection (ATCC, cat. #TIB.67, Manassas, VA, USA) cultured in growth media containing DMEM with 10% FBS with 50 IU/ml of Penicillin and 50 mcg/ml of streptomycin (Gibco-BRL) in an atmosphere of 5% CO_2_ and 95% air at 37°C. These macrophages were cocultured with MSCs obtained from mouse BM as mentioned previously. Coculture between these macrophages and MSCs was carried out using 4 *μ*m pore size polycarbonate membrane (Millipore, Burlington, MA, USA). Macrophages were seeded into a 6-well plate at a concentration of 2 × 10^5^ cells per well with 3 ml of growth media. MSCs were plated into the transwell insert at a concentration of 5 × 10^4^ cells per insert with 2 ml of MSCs growth media and the insert was placed into the 6-well plate. Macrophages and MSCs were cocultured for 7–10 days and inserts containing MSCs were removed from the 6-well plate and macrophages were collected with cell scraper. The single suspension cells (1 million cells/100 *µ*l PBS) were stained with mouse seroblock FcR blocker from Bio-Rad Laboratories (#BUF041A, Hercules, CA) for 20 min followed by staining with fluorescent antibodies for in dark. After 30 min-stained cells were washed with PBS, fixed with fixation buffer, and subjected to FACS analysis to determine the polarization of macrophages while cell pellets were used for protein extraction for western blot analysis. Macrophages cultured without coculture with MSCs taken as control cells. Fluorescent antibodies FITC-antimouse F4/80 (#123108) for macrophages and PE antihuman CD163 (#333605) for M2 macrophages were obtained from BioLegend (San Diego, CA, USA) while APC antimouse iNOS (#17-5920-82) for M1 macrophages purchased from ThermoFisher Scientific. The quantities of the antibodies and respective isotype controls were used as suggested by the manufacturing companies according to the protocols. The FACS analysis was carried out as mentioned above.

### 2.4. Endometriosis Induction and MSC Infusion

Endometriosis was induced surgically in mice, as previously described [26], with minor modification. Briefly, uterine horns were extracted from a donor mouse. Each horn was opened longitudinally and sectioned in half horizontally introducing a total of four sections per uterus. For transplantation, experimental recipient mice were anesthetized by inhalation of isoflurane (2.5 L/min) in conjunction with oxygen (1.5 L/min). Two uterine segments were sutured to each side (right and left) of the parietal peritoneum using 5-0 polyglactin suture (Vicryl), approximately 1 cm apart. The peritoneum and skin were closed with 4-0 polyglactin suture. Sham surgeries were performed for the control group, using the same surgical procedure without the introduction of extraneous uterine tissue. Development of the model was confirmed postperfusion by opening the abdominal cavity and confirming the presence of endometriotic lesions. Mice were divided into vehicle-(PBS) and MSCs-infusion groups (*N* = 8 per group). On Day 0 and Day 30 after endometriosis surgery, mice in the MSC-infusion group were retro-orbitally injected with 2 × 10^5^ MSCs in 100 *µ*l of PBS. Retro-orbital method is a reliable intravenous method that can easily allow larger numbers of BM cells into circulation. At 60 days of postendometriosis surgery, lesions were collected, and the volume was calculated using formula *V* = (1/2)*r*_1_ *r*_2_(*r*_1_ and *r*_2_ are radii,  *r*_1_ < *r*_2_) [27].

### 2.5. Transplantation of Bone Marrow Conditioned by GFP-MSCs

Bone marrow transplantation (BMT) was performed, as previously described [28, 29]. Briefly, female C57BL/6 mice were irradiated with two doses of 4.8 gray 3 hr apart and randomized into two groups (*N* = 8 per group): BMT without MSC conditioning was used as a control and MSCs + BMT as the experimental group. The BMT group received 1 × 10^7^ unfractionated BM cells while MSC + BMT group received 2 × 10^5^ cultured GFP-MSCs along with 1 × 10^7^ BM cells in 150 *µ*l PBS retro-orbitally within 1 hr of the second irradiation dose. At 3 and 30 days after injection, the donor MSC engraftment in BM was estimated by flow cytometry. Prior to endometriosis induction surgery, recipient mice were allowed a period of 30 days to recover from the BMT and irradiation. Endometriosis was induced as previously described. After surgery, estradiol (5 *µ*g/kg/day) was administered subcutaneously until the end of the study (1, 3, 5, or 60 days after surgery) [29].

### 2.6. Quantitative Real-Time Polymerase Chain Reaction (qRT-PCR)

Total RNA was isolated from BM or endometriotic lesions using TRIzol™ reagent followed by purification using RNeasy® spin columns. qRT-PCR was performed with the cDNA using iQ™ SYBER® Green Supermix (Bio-Rad) with specific primers for each gene. Gene expression was normalized to the expression of GAPDH. Relative mRNA expression was calculated by 2^−*ΔΔ*Ct^ method. The primer sequences used are given in Table 1.

### 2.7. Immunohistochemistry (IHC) and Immunofluorescence (IF)

Tissue from endometriotic lesions was fixed in 4% paraformaldehyde, embedded in paraffin, and cut into 5 *µ*m sections. After antigen retrieval followed by blocking, tissue sections were incubated at 4°C overnight with anti-F4/80 primary antibody (#MA1-91124, Invitrogen, Carlsbad, CA; 1 : 200) or antineutrophil elastase (NE) primary antibody (#ab68672, Abcam, Cambridge, UK; 1 : 200). Appropriate secondary antibodies and detection reagents were supplied by the Vectastain® Elite ABC-HRP kit (Vector Laboratories, Burlingame, CA, USA) and ImmPACT® DAB Substrate (Vector Laboratories).

For IF and colocalization studies, blocking was performed with 10% donkey serum (Vector Laboratories) for 1 hr. Sections were incubated with the following primary antibodies at 4°C overnight: goat anti-GFP antibody (#ab6673, Abcam, 1 : 1000), rat anti-F4/80 (MA1-91124, Invitrogen, 1 : 200), rabbit anti-iNOS antibody (#ab3523, Abcam; 1 : 100), and rabbit anti-CD206 antibody (#ab64693, Abcam, 1 : 500). The secondary antibodies: Alexa Fluor 568-conjugtated donkey antigoat (#A11057, Thermo Fisher Scientific), Alexa Fluor 647-conjugtated donkey antirat (#ab150155, Abcam), and Alexa Fluor 488-conjugtated donkey antirabbit (#A21206, Thermo Fisher Scientific) were all used in 1 : 200 dilution. Sections were mounted under coverslips using Vectashield® Antifade Mounting Medium with 46-diamidino-2-phenylindole (DAPI) (Vector Laboratories). Visualization of the slides was performed using a laser scanning confocal microscope Leica SP5 (Leica Microsystems, Wetzlar, Germany) and LAS-X software (Leica Microsystems). For immunocytochemistry, cultured MSCs were fixed in 4% PFA for 10 min, followed by the procedure as described above for IF without antigen retrieval.

### 2.8. Image Quantification and Analysis

Quantification of F4/80 or NE positive cells in lesions was performed after IHC staining. The total number of cells that stained positive in lesions was counted manually and expressed as a percentage of the total number of nucleated cells. For quantification of M1 macrophages (F4/80^+^iNOS^+^) and M2 macrophages (F4/80^+^/CD206^+^), 16 high-power confocal microscopy fields (eight HPFs from each of two sections per animal) were assessed. The number of M1 and M2 macrophages was counted and expressed as a percentage of F4/80^+^ cells.

### 2.9. Western Blot Analysis

Macrophages (J744 cell line) were cocultured for 7–10 days with and without BM-derived MSCs *in vitro* and lysed in cell lysis buffer (Cell Signaling, Danvers, MA, USA) with 1 mM PMSF (Sigma–Aldrich) with 1 mM NaF (Sigma–Aldrich). In total, 4x sodium dodecyl sulfate–polyacrylamide gel electrophoresis (SDS–PAGE) loading buffer (Bio-Rad Laboratories) was added to the sample, which was then boiled at 95°C for 3–5 min. Samples (10 *μ*g protein) were then loaded onto 4%–20% SDS-PAGE gels (Bio-Rad Laboratories) and run at 100 V for 1 hr. Protein was then transferred onto PVDF membranes and incubated in 5% bovine serum albumin (Sigma–Aldrich) for blocking. Membranes were then incubated in primary antibodies at a dilution of 1 : 1,000 for anti-iNOS (cat. #18985-1-AP, Proteintech Group Inc. Rosemont, IL, USA), and anti-CD163 (cat. #PA5109340, ThermoFisher Scientific, Waltham, MA, USA). Anti-*β*-actin (dilution 1 : 5000, ThermoFisher Scientific) was used as the housekeeping protein. Membranes were washed in TBS-T and then incubated in secondary antibody conjugated to horse-radish peroxidase goat antimouse IgG (dilution 1 : 5000, Jackson Immuno Research Laboratories Inc., West Grove, PA, USA). Membranes were then washed in TBST-T and treated with chemiluminescence reagent clarity Max Western ECL substrate solutions A and B (1 : 1 ratio, Bio-Rad Laboratories, Hercules, CA, USA) and then visualized using an Amersham 680 imager. The density of protein bands was assessed by the ImageJ software, and values were normalized to the densitometric values of *β*-actin. Western blots were run twice with duplicate samples.

### 2.10. Statistical Analysis

Data were analyzed using GraphPad Prism 9.0 (GraphPad Software, LA Jolla, CA). An unpaired Student's *t*-test for percentage of labeled cells (PLC), lesion volume, and qRT-PCR data was used to determine statistical significance. Nonparametric Kruskall–Wallace test was performed for ratio and densitometry data. Data were expressed as mean ± standard error (SEM).

## 3. Results

### 3.1. Characterization of Bone Marrow Derived MSCs

**Figure 1:**
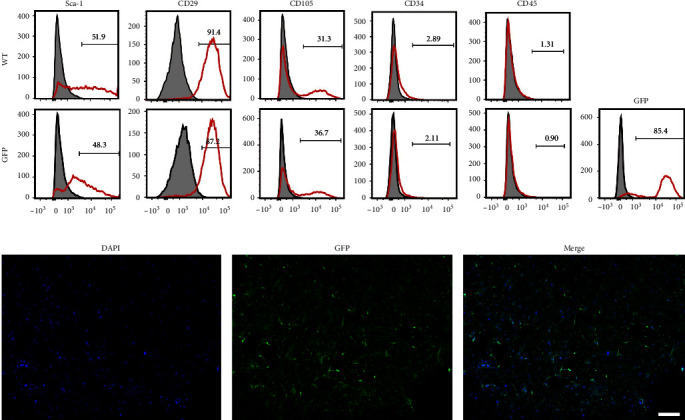
Cultured GFP-MSCs are phenotypically similar to primary wild-type MSCs. (a) Flowcytometric analysis demonstrates that GFP-MSC are negative for CD45 and CD34, and positive for CD29, CD105, and Sca-1, similar to wild-type MSC. Experiments were performed three times using unique samples and in duplicate. (b) GFP-MSCs uniformly express GFP within the cytosol. Scale bar: 200 *µ*m.

We have previously demonstrated the isolation of MSCs that are multipotent [9, 30] and able to differentiate into differentiate into adipocytes, osteocytes, and chondrocytes in mice [9]. Here, FACS analysis of MSCs similarly isolated from BM of both wild-type (no GFP) as well as mice expressing GFP expressed of MSCs markers including CD29, CD105, and Sca-1, and the absence of hematopoietic cell markers CD45 or CD34. Both WT and GFP-MSCs showed a similar expression pattern of these cell surface markers, as shown in Figure 1(a). MSCs expressing GFP were confirmed by immunocytochemistry before use (Figure 1(b)).

### 3.2. Direct MSCs Infusion Did Not Affect Lesion Growth

**Figure 2:**
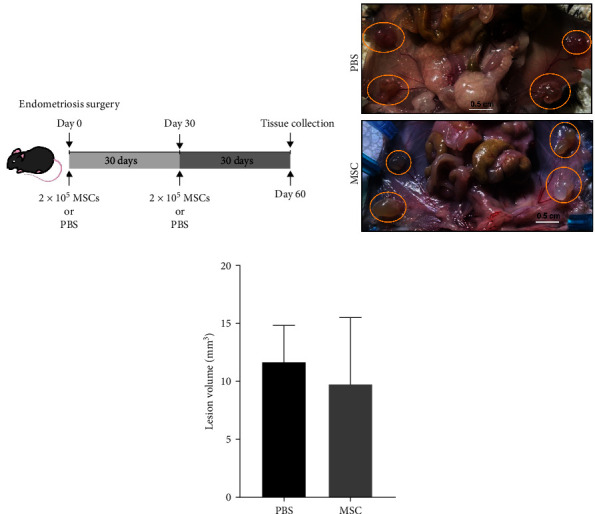
Endometriosis was not affected by direct MSC administration. (a) Schematic diagram showing timeline for MSC or vehicle administration and tissue collection from endometriosis mice (*N* = 8). MSCs were injected retro-orbitally on Day 0 and 30 after grafting uterine fragments into the peritoneal cavity. (b) Images showing lesions formation in mice administered PBS or MSCs (*N* = 8 each group). No significant differences were noted. (c) Lesion volume was assessed on Day 60. Results are expressed as mean SEM of total volume of lesions in mm^3^ (*p* = 0.14).

To investigate whether MSCs could affect the endometriotic lesion development, 2 × 10^5^ MSCs were injected retro-orbitally into mice with endometriosis on Day 0 and Day 30 after induction of endometriosis by surgery, as shown in Figure 2(a). Lesion formation and size in both PBS and MSCs treated groups are shown in Figure 2(b). The lesion size varied from 0.6 mm to 1 cm. In both groups, each mouse developed four lesions: two on right side and two on left side of the peritoneal cavity corresponding to the sites of initial transplantation. Evaluation of lesion volume showed that there were no significant differences between PBS and MSCs injected groups, as shown in Figure 2(c). GFP-expressing MSCs were absent from lesions, suggesting that the MSCs are not directly affecting endometriotic lesions.

### 3.3. In Vitro Coculture of Mouse Macrophages with MSCs Leads to Increased M2 and Decreased M1 Macrophages

**Figure 3:**
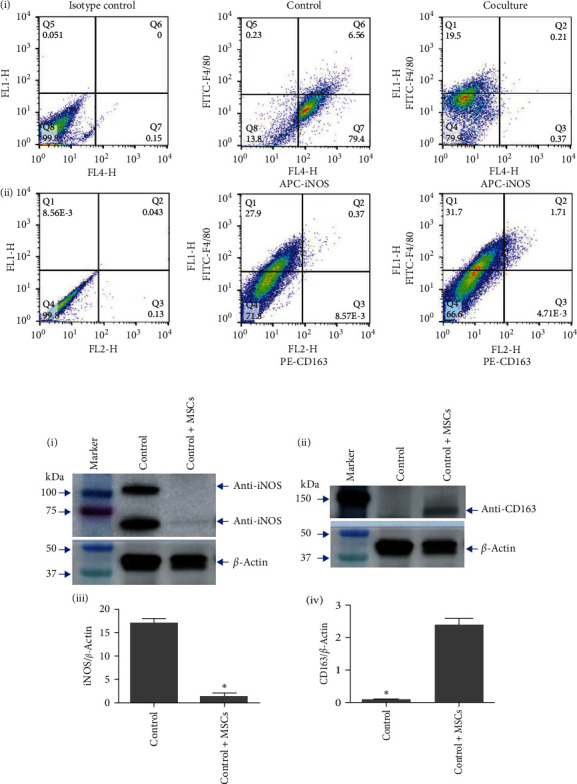
MSC program macrophage M1/M2 *in vitro*. (a) FACS analysis of macrophages cell line with cocultured with BM-derived MSCs. (i) M1 macrophages (iNOS^+^) were reduced by 30-fold in MSC cocultured cells compared to controls while (ii) M2 macrophages (CD163^+^) increased by fivefold. Experiments were performed three times each time in duplicate. (b) Western blot analysis of iNOS and CD163 showed protein levels of iNOS (i) were decreased while CD163 (ii) was increased in macrophages cocultured with MSCs from BM compared to macrophages cultured alone. The densitometry analysis of protein band ratios of iNOS and CD163 to *β*-actin are shown in bar graph (iii) and (iv), respectively. Protein levels of iNOS are significantly reduced while CD163 levels increased significantly in cocultured cells compared to macrophages alone. Each bar represents the mean ± SEM of two separate blots and each sample run in duplicate.  ^*∗*^*p* < 0.05 between macrophages alone vs. coculture with MSCs.

We next determined the direct effect of MSCs on BM-derived immune cells *in vitro*. We tested whether MSCs would have an effect on undifferentiated mouse macrophage cell line. Macrophage cells were cultured for 7 days with (coculture) and without MSCs derived from mouse BM. MSCs were plated in inserts while macrophages in 6-well plate as mentioned in methods. PE anti-CD163 and APC anti-iNOS antibodies used for staining M1 and M2 macrophages as markers, respectively, while FITC anti-F4/80 used for macrophages. FACS data analysis demonstrated a significant decrease in M1 by 30-fold and increase in M2 by fivefold macrophage populations after cocultured with MSCs compared to macrophages alone, as shown in Figure 3(a) (i) and (ii). The FACS results are further confirmed by measuring protein levels of iNOS and CD163 in macrophages cocultured with MSCs. Western blot analysis revealed that protein levels of iNOS were downregulated while CD163 was upregulated, as shown in Figure 3(b) (i) and (ii), respectively. The protein bands were normalized to *β*-actin protein levels by densitometry using ImageJ software. Analysis showed that protein levels of M1 marker iNOS was decreased by 18-fold (*p* = 0.005) while M2 marker CD163 levels increased by twofold (*p* = 0.007) in macrophages coculture with MSCs compared to control macrophages (Figure 3(b) (iii) and (iv)).

### 3.4. MSCs Program BM at the Time of BMT

**Figure 4:**
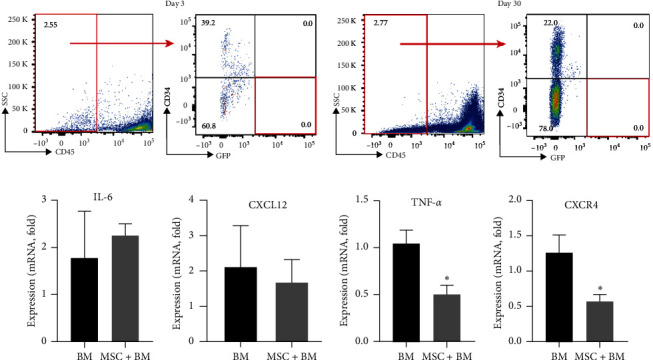
Conditioning of BM with MSCs and cotransplantation did not result in MSC engraftment of BM however did program BM gene expression. (a) Flowcytometric analysis of BM cells after GFP-MSC cotransplantation demonstrated the complete absence of MSCs (CD45-CD34-GFP+) on Day 3 or Day 30 (*n* = 8). (b) Differential expression (mRNA levels) of CXCL12, CXCR4, IL6, and TNF-*α* in BMCs were analyzed by qRT-PCR at 30 days after BMT expressed relative to transcript level in BMCs of control mice not receiving transplant. Each bar represents the mean ± SEM of two individual experiments and each was performed in triplicates.  ^*∗*^*p* < 0.05 BM vs. BM + MSCs (*N* = 8/group).

To assess the effect of the MSCs on BM, BM was conditioned and cotransplanted with GFP-MSCs cultured *in vitro*. FACS analysis showed that there were no donor GFP expressing MSCs found in recipients' BM after BM transplantation. No GFP positive cells were detected in BM at Day 3 or 30 after MSCs cotransplantation, as shown in Figure 4(a). Transplantation of MSCs alone resulted in death within 14 days after irradiation, further indicating no direct role of MSCs in restoring the BM. Though the donor GFP-MSCs were undetectable in the BM, several alterations in BM were seen in the cotransplantation group. TNF-*α* and CXCR4 mRNA levels were significantly reduced in BM (*p* = 0.02 and 0.03, respectively) after cotransplantation but not BM transplant alone, as shown in Figure 4(b). These results suggest that, although cultured MSCs did not permanently engraft BM, they could affect the BM microenvironment during a transient exposure and a role for MSCs in remodeling of BM.

### 3.5. BMT Conditioning and Cotransplantation with MSCs Inhibited Lesion Development and Macrophage Infiltration into Lesions

**Figure 5:**
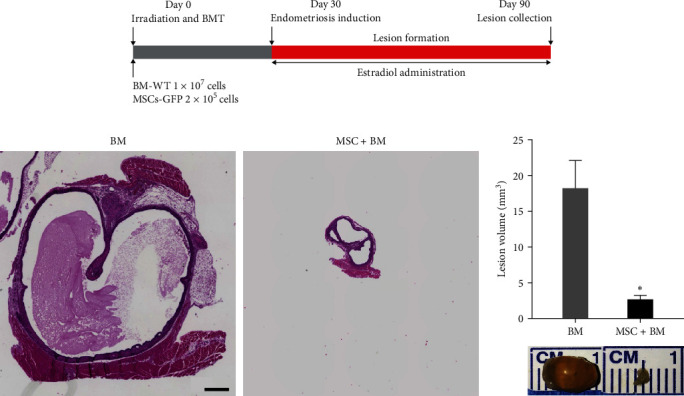
Cotransplantation of MSCs with BM reduced the lesion size in mice. (a) Schematic diagram showing timeline for BMT performed with or without MSCs into irradiated mice (*N* = 8 per group). Endometriosis was induced on Day 30 after BMT and tissue was collected after 60 days. (b) Representative images of H&E stained endometriotic lesions from BM and MSC + BM groups (*N* = 8). Scale bar: 300 *µ*m. (c) Photograph showing reduced lesion size in MSC + BM group compared to BM transplanted group. Lesion volume was assessed on Day 60 after surgery. Data (*N* = 8) are expressed as means ± SEM.  ^*∗*^*p* < 0.0001.

**Figure 6:**
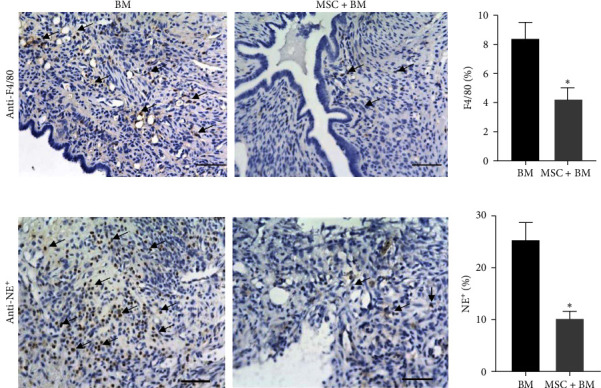
Macrophages and neutrophils infiltration into lesions was reduced in the MSC + BM transplanted group compared to the group not condition by MSCs. (a, b) Representative IHC images showing fewer cells stained using anti-F4/80 antibody (macrophages) or antineutrophil elastase (NE) antibody (neutrophils), respectively, in the lesions from BM and MSC + BM groups. Scale bar: 50 *µ*m. Bar graphs showing quantitative analyses of F4/80 as well NE positive cells that were significantly reduced in the MSC + BM group compared to the BM group (*N* = 8,  ^*∗*^*p* < 0.05).

We next determined if the MSCs entrained BM would impact endometriosis growth. The experimental schematic diagram is shown in Figure 5(a). Surprisingly, we found that cotransplantation of MSCs along with BMT after irradiation of mice resulted in more than 80% reduction in the lesion volume compared to BMT alone, as shown in Figure 5(b). We found an approximately twofold difference in macrophage (*p* = 0.03) and neutrophil (*p* = 0.02) infiltration into the endometriotic lesions between the groups undergoing BMT with and without MSCs pretreatment/cotransplantation. Data for all lesions were shown in *Supplementary 1*. Using IHC staining, we demonstrated that F4/80^+^ macrophages (Figure 6(a)) and NE^+^ neutrophils (Figure 6(b)) were recruited in smaller numbers into lesions in the MSCs + BMT cotransplantation group compared to the BM control group. The negative isotype control is shown in *Supplementary 2*. The percentage of the infiltration of macrophages and neutrophils is 4% and 10%, respectively, in the MSC-treated BM group compared to 9% and 26%, respectively, in the control BM group. The isotype negative controls were shown in *Supplementary 2*.

### 3.6. MSCs Inhibited M1 Macrophages Polarization in Endometriotic Lesions

**Figure 7:**
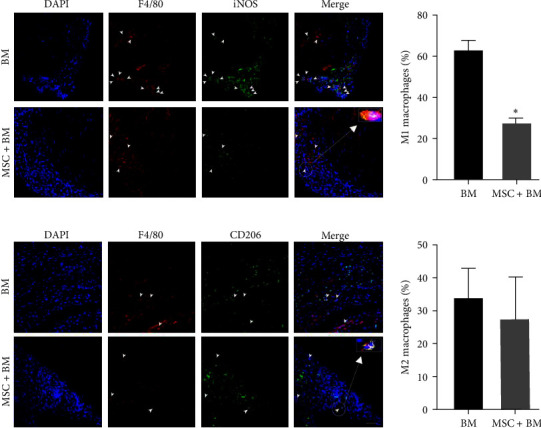
Polarization of M1 macrophages infiltrated into lesions from MSC + BM cotransplanted mice. (a) Fluorescence confocal microscopy analysis of M1 macrophage polarization. Representative images of IF showing DAPI, F4/80^+ve^, iNOS^+ve^, and merged images. Nuclei were stained using DAPI and are shown in blue. F4/80 marks macrophages while iNOS is a marker of M1 macrophages. Bar graphs showing quantitative analyses of M1 macrophage polarization (iNOS^+ve^ cells) which were significantly reduced in MSCs + BM cotransplantation group compared to BM group (*N* = 8, *p* < 0.05). (b) Fluorescence confocal microscopy analysis of M2 macrophage polarization. Representative images of IF showing DAPI, F4/80^+ve^, CD206^+ve^, and merged images. Nuclei were stained by DAPI and are shown in blue. Bar graph showing no significant difference between the groups in M2 macrophage polarization (*N* = 8).

To investigate the response of macrophages in ectopic uterine tissue with or without MSCs cotransplantation, we performed IF double staining on tissue sections from lesions from both groups. F4/80 was used as a specific macrophage marker, iNOS and CD206 were used as markers for M1 and M2 macrophages, respectively. IF staining showed that M1 macrophages in ectopic tissues (lesions) were significantly reduced in the MSCs + BMT cotransplantation group at Day 5 after surgery compared to BMT group (Figure 7(a)). No significant differences were observed in M2 macrophages between the groups (Figure 7(b)). The ratio of M1/M2 macrophages was reduced by 53% (*p* = 0.03).

## 4. Discussion

In this study, we describe the role of MSCs and their ability to program BM and demonstrate that this programing can affect endometriosis. MSC programing of BM results a significant reduction in lesion growth and decreased M1 macrophage infiltration in a murine model of endometriosis. Our results imply that BM-derived immune cells are an important, yet modifiable, determinant of endometriosis. BM programing has an important and previously unrecognized role in the pathophysiology of endometriosis.

MSCs from BM can engraft endometriosis and contribute to lesion growth. Blocking CXCR4 inhibits MSC engraftment into lesions and leads to regression of disease [29, 31, 32]. However, in this study, the exogenous MSCs did not engrafted the BM or lesion, rather they had a transient effect at the time of BM transplant that altered the endogenous BM. FACS data analysis of MSCs showed that there were no GFP-labeled donor MSCs in the BM of recipient mice either on Day 3 or 30 after transplantation. This may be due to short-lived MSCs that are unable to engraft after intravenous infusion [33, 34]. Secretion of various bioactive molecules that alter the tissue microenvironment is believed to be the main mechanism by which MSCs achieve their therapeutic effect [35]. In our model, even direct MSCs transplant into nonirradiated mice did not influence endometriotic lesion development and growth. In contrast, lesion volume was dramatically reduced in the mice that received BM which was conditioned and cotransplanted with MSCs following irradiation, compared to those receiving BM alone. Despite the absence of the GFP-labeled donor MSCs, endometriotic lesion development was reduced significantly in the MSCs + BM transplant group compared to BM group. MSCs have a role in BM remodeling, inducing BM memory through tropic factors rather than by long-term engraftment. MSCs have powerful regulatory properties, inducing long-lasting effects in cells well after transient contact or exposure. The programing of BM by MSCs likely also has long-term effects on the immune system and other inflammatory diseases in addition to endometriosis.

Macrophage-induced inflammation appears to be an important driver of endometriosis. Endometriosis is accompanied by an increase in peritoneal macrophages and a profound systemic inflammatory response [13, 36]. Differences in BM and BM-derived cells likely have a central role in the establishment and progression of endometriosis. Rodent models for endometriosis have demonstrated that macrophage depletion inhibits development and growth of endometriotic implants [12, 37–39]. Similar to these reports, our results showed that the macrophage population in endometriosis was reduced, specifically M1 macrophages were significantly reduced in MSC + BM group compared to the BM group. These results suggest that persistence of inflammation caused by M1 macrophages could contribute to lesion development while decrease in M1 macrophage population reduces inflammation thereby inhibit the lesion growth. Our findings are consistent with known involvement of BM-derived cells, especially macrophages, in the development of endometriosis [15, 20].

Lange et al. [40] reported that gene expression profile in BM changed after MSCs infusion. These results agree with our data as we found that mRNA levels of CXCR4 and TNF-*α* are reduced significantly in MSC + BM group compared to the BM group. CXCR4 is a chemokine receptor which is expressed in leukocytes, macrophages, and BM stem/progenitor cells [41–43] and involved in immune cells or stem/progenitor cells chemotaxis in several inflammation conditions [44, 45], including endometriosis [31, 46]. Our previous study showed that pharmacological antagonism of CXCR4 led to the regression of endometriotic lesions in mice through blocking recruitment of BM-derived cells to the lesions [31]. The reduction of CXCR4 expression in BM cells could contribute to decreased inflammatory macrophage engraftment and lesion regression.

TNF-*α* is a proinflammatory cytokine, and, while its function in BM is controversial, it has been clearly implicated in the pathophysiology of endometriosis [47]. We found decreased TNF-*α* expression in BM after MSC cotransplantation. TNF-*α* is produced by M1 macrophages in the peritoneal fluid of women with endometriosis and correlates with lesion number and size [48, 49]. Similarly, monocytes from patients with endometriosis produce increased levels of TNF-*α* [50]. Reduced levels of TNF-*α* in BM may indicate a reduction in the propensity of BM to generate TNF-*α* producing M1 macrophages that drive endometriosis.

In conclusion, we found that MSCs alter BM programing and induce BM memory that prevents endometriotic lesion development. MSCs have powerful regulatory properties, inducing long-lasting effects in cells well after transient contact or exposure. BM programing by MSCs appears to play a crucial role in endometriosis. Differences in BM and BM-derived cells likely account for some of the variation in human susceptibility to this disease. BM remodeling is a novel target for the treatment of endometriosis.

## Figures and Tables

**Table 1:** Primer sequences used for qRT-PCR.

Gene	Forward	Reverse
CXCL12	5ʹ TGCATCAGTGACGGTAAACCA 3ʹ	5ʹ CACAGTTTGGAGTGTTGAGGAT 3ʹ
CXCR4	5ʹ ACGGCAACCTCATGAACCA 3ʹ	5ʹ GGAAACGGCTCCCCTTGA 3ʹ
IL6	5ʹ CTGCAAGAGACTTCCATCCAG 3ʹ	5ʹ AGTGGTATAGACAGGTCTGTTGG 3ʹ
TNF-*α*	5ʹ ATGGCCCAGACCCTCACACTCA 3ʹ	5ʹ TGGTGGTTTGCTACGACGTGGG 3ʹ
GAPDH	5ʹ GCCTGCTTCACCACCTTCTT 3ʹ	5ʹ ATGGCCTTCCGTGTTCCTAC 3ʹ

## Data Availability

Data used in this study are available with Dr. Hugh Taylor and Dr. Ramanaiah Mamillapalli upon request.

## Supplementary Materials

Supplementary 1 Lesion volume for all mice (*N* = 8).

Supplementary 2 Representative images of isotype controls for anti-F4/80 antibody and anti-elastase antibody by immunostaining. Tissue sections were immune stained by respective isotype control antibodies.

Supplementary 3 Flowcytometry data for Figure 1.

Supplementary 4 Lesion volume of mice in PBS and MSC groups.
