# Supplementary material for: Donor Mesenchymal Stem Cells Program Bone Marrow, Altering Macrophages, and Suppressing Endometriosis in Mice
Source: Stem Cells Int. 2023 Jul 28;2023:1598127. doi: 10.1155/2023/1598127 (PMC10403325; doi:10.1155/2023/1598127)
Supplement: Supplementary 1 — Lesion volume for all mice (N = 8). [file 1598127.f1.docx]

**Supplemental Table 1**

| **Mice #** | **Lesion #** | **BM** | **MSC+BM** |
| --- | --- | --- | --- |
|  |  | Lesion Volume (mm^3^) | Lesion Volume (mm^3^) |
| 1 | 1 | 45 | 0.625 |
|  | 2 | 46 | 10.125 |
|  | 3 | 5.875 | 4.75 |
| 2 | 4 | 27.375 | 0.625 |
|  | 5 | 6.75 | 5.25 |
|  | 6 | 6.23 | 2.25 |
|  | 7 | 21.5 | 0.375 |
| 3 | 8 | 5.21 | 1.125 |
|  | 9 | 13.625 | 3.25 |
|  | 10 | 18 | 1.75 |
| 4 | 11 | 6.5 | 5.25 |
|  | 12 | 6.25 | 2.25 |
| 5 | 13 | 7.25 | 3 |
|  | 14 | 6.26 | 2.125 |
| 6 | 15 | 13.625 | 0.750 |
|  | 16 | 4.63 | 3.750 |
| 7 | 17 | 6.33 | 1.875 |
|  | 18 | 43.5 | 0.876 |
| 8 | 19 | 20.65 | 3.25 |
|  | 20 | 5.75 | 0.5 |

**Supplemental Table 1:** Lesion volume for all mice (N=8)
