# Supplementary figures and images for: Donor Mesenchymal Stem Cells Program Bone Marrow, Altering Macrophages, and Suppressing Endometriosis in Mice
Source: Stem Cells Int. 2023 Jul 28;2023:1598127. doi: 10.1155/2023/1598127 (PMC10403325; doi:10.1155/2023/1598127)

## Supplemental Figure 1

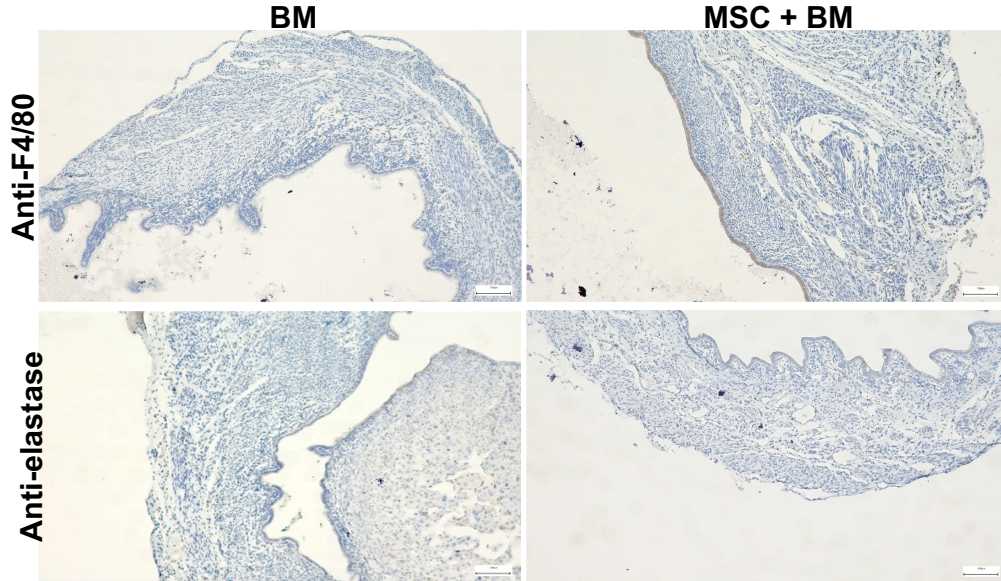

Supplement: Supplementary 2 — Representative images of isotype controls for anti-F4/80 antibody and anti-elastase antibody by immunostaining. Tissue sections were immune stained by respective isotype control antibodies. [file 1598127.f2.pdf]
