# Supplementary material for: Donor Mesenchymal Stem Cells Program Bone Marrow, Altering Macrophages, and Suppressing Endometriosis in Mice
Source: Stem Cells Int. 2023 Jul 28;2023:1598127. doi: 10.1155/2023/1598127 (PMC10403325; doi:10.1155/2023/1598127)
Supplement: Supplementary 3 — Flowcytometry data for Figure 1. [file 1598127.f3.docx]

**Supplemental Table 2**

**Supplemental Table 2**: Flowcytometry data for figure 1

|  | **WT**  **(freq. of parent)** | **GFP-MSC**  **(freq. of parent)** | **P-value** |
| --- | --- | --- | --- |
| SCA1 | 51.95 | 48.65 | 0.61 |
| CD29 | 91.7 | 87.6 | 0.78 |
| CD105 | 32.15 | 35.85 | 0.61 |
| CD34 | 3.05 | 2.31 | 0.11 |
| CD45 | 1.205 | 0.95 | 0.16 |

Sample number: N=8
