# Supplementary material for: Donor Mesenchymal Stem Cells Program Bone Marrow, Altering Macrophages, and Suppressing Endometriosis in Mice
Source: Stem Cells Int. 2023 Jul 28;2023:1598127. doi: 10.1155/2023/1598127 (PMC10403325; doi:10.1155/2023/1598127)
Supplement: Supplementary 4 — Lesion volume of mice in PBS and MSC groups. [file 1598127.f4.docx]

**Supplemental Table 3**

| Mice Number | PBS Lesion volume (mm^3^) | MSC Lesion volume (mm^3^) |
| --- | --- | --- |
| 1 | 12.5 | 15 |
| 2 | 12.125 | 5.75 |
| 3 | 25.75 | 8.375 |
| 4 | 10.5 | 15.625 |
| 5 | 5 | 18.375 |
| 6 | 3.85 | 6.25 |
| 7 |  | 2.375 |
| 8 |  | 6 |

**Supplemental Table 3:** Lesion volume of mice in PBS and MSC groups
